# Crosstalk between the Smad and the Mitogen-Activated Protein Kinase Pathways is Essential for Erythroid Differentiation of Erythroleukemia Cells Induced by TGF-β, Activin, Hydroxyurea and Butyrate

**DOI:** 10.4172/2329-6917.1000109

**Published:** 2013-04-22

**Authors:** Salem Akel, Daniel Bertolette, Francis W Ruscetti

**Affiliations:** 1 Leukocyte Biology Section, Laboratory of Experimental Immunology, Center for Cancer Research, NCI, Frederick, Maryland; 2 St. Louis Blood Bank and Cellular Therapy Laboratory, Cardinal Glennon Children’s Medical Center, St. Louis, MO

**Keywords:** Transforming Growth Factor-β, Activin, Hydroxyurea, Erythropoiesis, Smads, p38 MAP Kinase, Okadaic acid

## Abstract

The role of crosstalk between the Smad and the MAPK signaling pathways in activin-, transforming growth factor-β (TGF-β)-, hydroxyurea (HU) - and butyrate-dependent erythroid differentiation of K562 leukemic cells was studied. Treatment with all four inducers caused transient phosphorylation of Smad2/3 and MAPK proteins including ERK, p38 and JNK. Use of specific inhibitors of p38, ERK and JNK MAPK proteins, and TGF-β type I receptor indicated that differentiation induced by each of these agents involves activation of Smad2/3 and p38 MAPK, and inhibition of ERK MAPK. Also, treatment of cells with an inhibitor of protein serine/threonine phosphatase, okadaic acid (OA), induced phosphorylation of Smad2/3, and p38 MAPK, coincident with its induction of erythroid differentiation. Specific inhibition of TGF-β type I receptor kinase activity not only abolished TGF-β/activin effects but also prevented Smad2/3 activation and erythroid differentiation induced by OA, HU and butyrate. The TGF-β type I receptor kinase inhibitor blocked OA-induced differentiation but not p38 MAPK phosphorylation demonstrating that signals from both pathways are needed. As previously observed, addition of ERK1/2 MAPK inhibitors upregulated Smad2/3 phosphorylation and enhanced differentiation, but these effects were dependent on signals from the TGF-β type I receptor. These data indicate that activation of both Smad2/3 and p38 MAPK signaling pathways is a prerequisite to induce erythroid differentiation of erythroleukemia cells by activin, TGF-β, HU, OA and butyrate.

## Introduction

The transforming growth factor (TGF-β) family regulates most, if not all, mammalian cellular processes [1]. Two members, activins and TGF-βs, of the TGF-β family have many such effects on hematopoiesis, when appropriate in context, from stem cell quiescence, progenitor cell growth inhibition, stimulation of differentiation, mature cell function and cell death. Ligand binding induces phosphorylation and activation of type I receptor signaling by the type II receptor kinase. Smad2 and Smad3, the principal receptor-activated proteins involved in activin/ TGF-β signaling, are activated directly by TGF-β type I receptor kinase then translocate to the nucleus in complex with a common co-Smad, Smad4, to regulate transcription of target genes [2-4]. Smad6 and Smad7 are inhibitory Smads that interact with all TGF-β type I receptors antagonizing activin/ TGF-β signaling [1-7].

The response of TGF-β signaling is cellular type- and context-dependent [8,9]. This could involve different receptor complexes [10], cross talk with other signaling pathways, different transcription factor activities and genetic changes in cancers. Many signaling pathways interact with activin/ TGF-β signaling [1-4]. The mitogen-activated protein kinase (MAPK) cascades, comprised mainly of the extracellular signal-regulated kinase (ERK), p38 MAPK, and the c-Jun N-terminal kinases (JNK) [11], have been shown to represent a major signaling pathway for TGF-β and activin independent of Smad activation [3,12-19]. In crosstalk between the Smad and MAPKs pathways, TGF-β dependent or independent induction of activated ERK MAPK can occur. Activated ERK by activation of the epidermal growth factor (EGF) receptor-Ras pathway and by Ca^2+^- calmodulin-dependent kinase II [20,21]. Phosphorylation of Smad2/3 by ERK MAPK imposes an inhibitory effect by attenuating their nuclear translocation [18,20,21]. In contrast, activation of ERK MAPK by hepatocyte growth factor had positive effects on Smad activation [18,22]. In the nucleus, an additional crosstalk between these signaling pathways of TGF-β and activin occurs [1-4]. Reciprocal regulation (positive or negative) of activated Smads and downstream substrates of JNK and p38 MAPKs, including c-Jun and ATF-2 (components of the AP-1 complex), has been shown [1-4,23-25]. This dual ability of the TGF-β type 1 receptor (TβR1) to independently activate signaling pathways which cross-talk can have profound effects on cellular processes.

TGF-β and activin both regulate different cellular events of hematopoiesis in a lineage specific manner [26-28]. Both cytokines stimulate erythroid differentiation, accompanied by hemoglobin synthesis, and TGF-β inhibited growth of early but not late erythroid progenitor cells [26,29-32]. Although erythroid differentiation induced by erythropoietin (Epo), hydroxyurea (HU) and sodium butyrate involves p38 activation and ERK1/2 inhibition [33-37], the requirement of MAPK signaling in TGF-β- and activin-induced erythroid differentiation remains obscure. The possible integration of these two pathways in erythroid differentiation was studied. Activin/ TGF-β- dependent erythroid differentiation requires activation of both Smad and p38 MAPK signals for optimal differentiation. Chemical induction of erythro-differentiation by HU and butyrate also resulted in phosphorylation of Smad2/3 and was TRI receptor dependent. Inhibition of ERK1/2 enhanced Smad2/3 phosphorylation and erythroid differentiation but this cross talk between the Smad and the ERK MAPK pathway was also TRI receptor dependent.

## Materials and Methods

### Cell culture and reagents

Erythroleukemia cells including K562, HEL and TF-1 were maintained in RPMI-1640 medium containing 10% fetal bovine serum (FBS). Interleukin (IL)-3 (10 ng/ml, Peprotech, Rocky Hill, NJ) was added to support the growth of TF-1 cells. To assay erythroid differentiation, cells (2×10^4^/ml) were cultured in serum-free media containing 10% BIT 900 (Stem Cell Tech.) with or without the addition of rh-activin A, rh-TGF-β1 (R &D systems, Minneapolis, MN, USA), HU, sodium butyrate (Sigma, St Louis, MO) and okadaic acid (LC Laboratories, Woburn, MA). To elucidate the role of each MAPK in the regulation of cell response, specific signal transduction inhibitors in dimethyl sulfoxide (DMSO) or DMSO alone were added to cultures one hour prior to cell treatment, as indicated. Inhibitors of ERK1, -2 (PD98059 and U0126), p38 (SB203580), and JNK1, -2, -3 (SP600125) MAPK (all used at 10 μM, 0.2 DMSO) were purchased from BioSource International, Camarillo, CA, USA. SB505124, a specific inhibitor of TβR1 that inhibits activation of both activin and TGF-β type I receptors (ALK4 and ALK5 as well as ALK7 respectively, 39) was provided by GlaxoSmithKline, (King of Prussia, PA). It was used at 20 uM (0.2% DMSO) with a control (0.2% DMSO).

### Evaluation of cell growth and differentiation

In the signaling studies on cells undergoing differentiation, cells were derived of serum overnight which reduces the signaling background at time zero due to factors in the serum, allowing the inhibitors to be more effective. Also, cells accumulate at G0/G1 allowing for more coordinated study on growth inhibition. A dose response of the differentiation inducers on K562 was performed to determine the optimal concentration for differentiation and cell signaling. Count of viable cells was determined using trypan blue staining. Formation of erythroid cells was monitored by flow cytometry using anti-glycophorin A (GPA) antibody (Immunotech, Marseille, France). The intracellular hemoglobin synthesis was monitored by staining of cells in suspension using 1% 2,7-diaminofluorene (DAF), 3% hydrogen peroxide and 5% acetic acid in Tris-HCl buffer (200 mmole/L; pH 7.0) [38].

### Evaluation of cell apoptosis

Cells grown in the presence and absence of activin A and TGF-β1 (as indicated) were harvested at different times and analyzed for apoptosis. Whole cell lysates were evaluated for the cleavage of the nuclear poly (ADP-ribose) polymerase (PARP) by western blotting as described below, using an anti-PARP antibody (Cell Signaling Tech., Beverly, MA, USA). Cells were also were examined for early apoptosis by evaluating the expression of the mitochondrial membrane protein APO2.7 using flow cytometric analysis of cells stained with PE-conjugated anti-APO2.7 antibody (Immunotech, Marseille, France) as previously described [39].

### Flow cytometric analysis

Treated cells, as indicated, were washed in 2% normal human AB serum in phosphate buffered saline (PBS), blocked with 5 μl of a mixture of normal human and mouse sera, washed, and incubated with 15 μl of either anti-GPA antibody conjugated with fluorescein isothiocyanate (FITC) (Immunotech) or an isotype control-FITC antibody (BD Laboratories, San Diego, CA) at 4°C for 30 minutes. After washing, cells were suspended in equal volumes of PBS and 4% paraformaldehyde. Analysis was carried out using a FACScan (BD laboratories) flow cytometer and FLOWJO software (Tree Star Inc., Palo Alto, CA, USA).

### Western blot analysis

Relative levels of proteins were assessed in whole cell lysates using the standard immunoblotting technique [32,40,41]. Smad signaling was evaluated using anti-Smad2 (Zymed Laboratories, South San Francisco, CA), anti-phospho-Smad2 and anti-Smad2/3 (Upstate Biotech. Lake Placid, NY), and anti-phospho-Smad3 (provided by Dr. Ed Leof, Mayo Clinic, Rochester, MN). Antibodies directed against MAP kinases [anti-p44/42, anti-phospho-p44/42 (r202/Tyr204), anti-p38, anti-phospho-p38 (Thr180/Tyr182), anti-SAP/JNK and anti-phospho-SAP/JNK (Thr183/Tyr185)] were obtained from Cell Signaling Tech. Briefly, cells were lysed in Triton X-100 lysis buffer (25 mM HEPES at pH 7.5, 150 mM NaCl, 10% glycerol, 5 mM EDTA, 1% Triton-X 100) containing phosphatase and protease inhibitors. Cell lysates were separated by SDS-PAGE and transferred onto an Immobilon-P membrane (Millipore). After blocking with 5% non-fat dry milk and incubation (2 hrs) with phosphoprotein specific primary antibody, blots were incubated (1 hr) with an appropriate secondary antibody (horseradish peroxidase-conjugated) and detected with the ECL system (Amersham Biosciences, UK).

The gel was then stripped and incubated with an antibody that recognizes the total protein of phospho-specific protein antibody.

### Statistical analysis

Results are expressed as a mean value + standard deviation (SD). The paired Student *t*-test value was used to determine the statistical significance of differences between means that was calculated from at least three independent experiments.

## Results

### Induction of erythroid differentiation/growth inhibition of human erythroleukemia cells by activin and TGF-β- requires activation of endogenous Smad2/3

**Figure 1:**
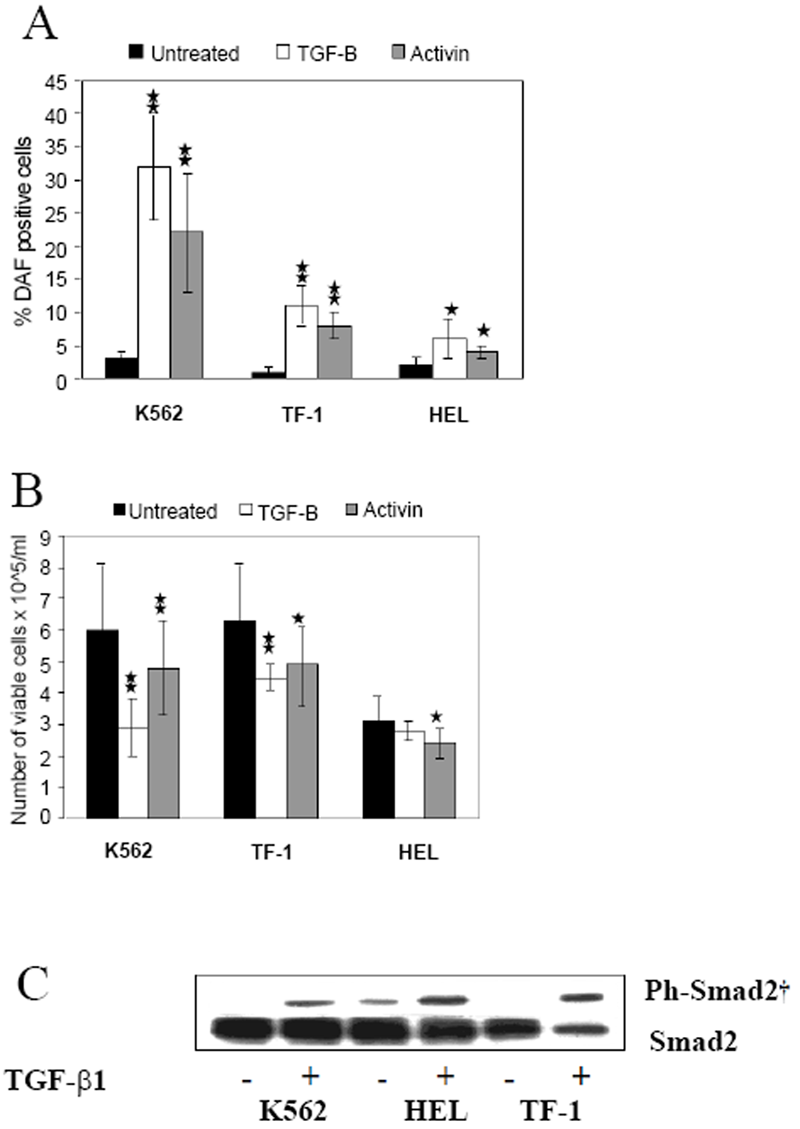
Differentiation response of erythroleukemia cells to TGF-β and activin treatment. K562, TF-1 and HEL cells (2×10^4^/ml) were cultured in serum free conditions without (-) and with (+) the addition of rh- TGF-β1 or rh-Activin-A (5 ng/ml each). After 6 days of culture, cells were collected and evaluated for their growth and intracellular hemoglobin synthesis. **A**. Percentage of Hb^+^ cells was calculated following DAF staining of cells in suspension and scoring at least 1000 cells. **B**. Total number of viable cells per ml was determined in same cultures after cell staining with trypan blue. **C**. In the same cultures, aliquots of cells were harvested after 1 hr of cytokine treatment and whole cell lysates were prepared as described in Materials and methods. Immuno-blotting with anti-phospho-Smad2 and anti-Smad2 was performed. Results are shown as the mean ± SD of four independent experiments. ** P<0.01 and *P<0.05

**Figure 2:**
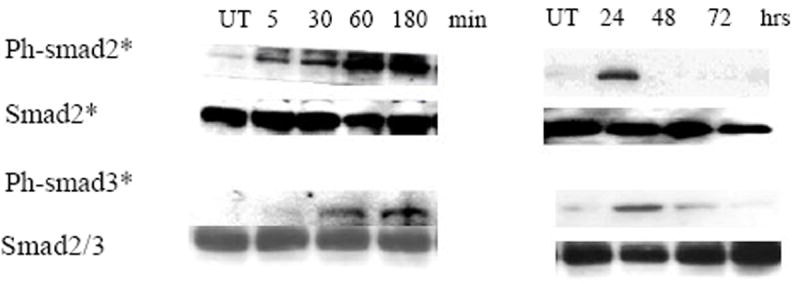
Time course of activation of Smad proteins by TGF-β1/activin. K562 cells were treated with 5 ng/ml of rh- **TGF-β**1, harvested at various time points (left: 5, 30, 60, and 180 minutes; right: 24, 48 and 72 hrs), lysed, and analyzed by immunoblotting. Membranes were probed with specific antibodies against the phosphorylated forms of the Smad proteins (Smad2 and Smad3). Protein-antibody reaction was detected by chemiluminescence. Same blots were stripped and hybridized with antibodies against Smad2 and Smad2/3 to evaluate the total corresponding proteins. Results are representative of 3 experiments. Similar results using antibodies against phospho-Smad3 and Smad2/3 proteins.

**Figure 3:**
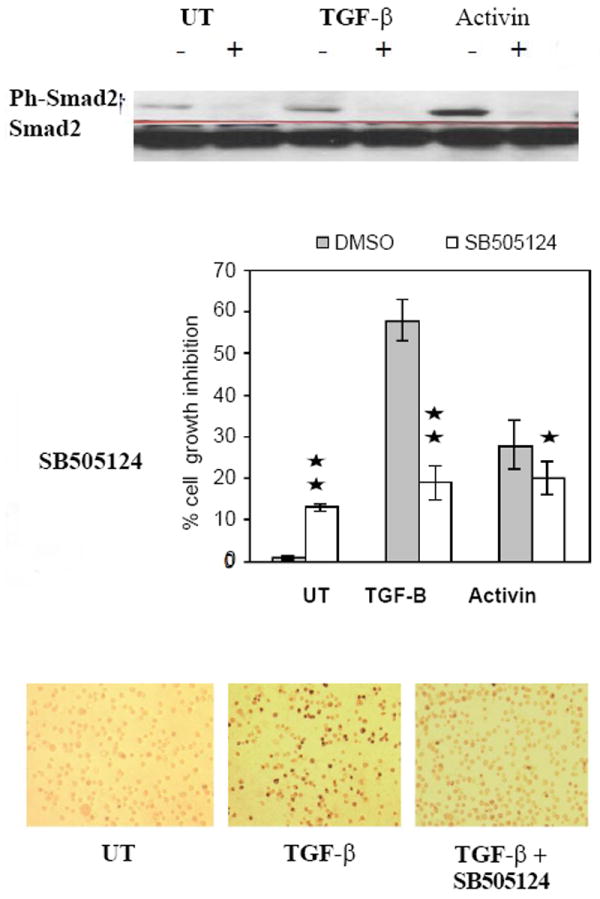
Inhibition of TGF-β type I receptor signaling blocked the response of K562 cells to TGF-β /activin treatment. Overnight-starved K562 (5×104/ml) cells were cultured for 1 hr with SB505124 (20 μM) in DMSO (+) and with DMSO alone (-). Cultures were split and continued in the absence (UT) and the presence of **TGF-β**1 and activin A (5 ng/ml). **A**. After 1 hr, 2 × 106 cells were harvested and subjected to immunoblot analysis using Anti-phospho-Smad2 antibody. Blots were stripped and reprobed with antibody that detects total Smad2 protein. **B**. Aliquots of the same treated cells were cultured for 5 more days. At this point, cells were evaluated for growth inhibition using counts of trypan blue negative viable cells and for the appearance of hemoglobinized DAF^+^ cells. **C**. Aliquots of the same treated cells were cultured for 5 more days. At this point, cells were evaluated for growth inhibition using counts of trypan blue negative viable cells. Results of A and C are representative of three experiments and results in B are mean values ± SD obtained from three independent experiments. Similar results were seen in cells treated with the same dose of activin A.

Activin A and TGF-β1 can inhibit the growth of erythroleukemia cell lines and stimulate erythroid differentiation, in a dose dependent fashion [29,30]. Similar results were seen on differential growth inhibition and increase in the proportion of hemoglobin positive (Hb^+^) DAF^+^ cells [38] after treatment of K562, TF-1 and HEL cell lines with rh-activin A or rh- TGF-β1 (Figures 1A and 1B). The greater growth inhibition coincided with greater erythroid differentiation. Phosphorylation of Smad2 was strongly induced following TGF-β (Figure 1C) and activin (data not shown) treatment in the three cell lines. The activation pattern of Smad2 and Smad3 by either cytokine was similar with rapid phosphorylation (beginning at 5 min and reaching a peak at 30-60 min) that lasted for 24 hours as shown in K562 (Figure 2). Smad3 activation lasted longer than Smad2. K562 cells were then treated with 20 M of SB505124, an inhibitor of phosphorylation of the TβR1 receptors (ALK4 and ALK5) upstream of Smad2/3 activation [42] (ALK 7 is also inhibited but responds to Nodal not TGF-β, activin). SB505124 not only prevented Smad2/3 phosphorylation (Figure 3A), but also blocked growth inhibition (Figure 3B) and suppressed erythroid differentiation (Figure 3C) induced by activin and TGF-β. The differentiation activin induced was 24% and reduced to undetectable by the inhibitor. Similarly differentiation induced by TGF-β went from 35% to undetectable. Thus, TGF-β receptor/Smad signaling is not defective in these erythroleukemia cells and is required for erythroid differentiation mediated by these ligands.

### Induction of erythroid differentiation precedes early apoptosis

**Figure 4:**
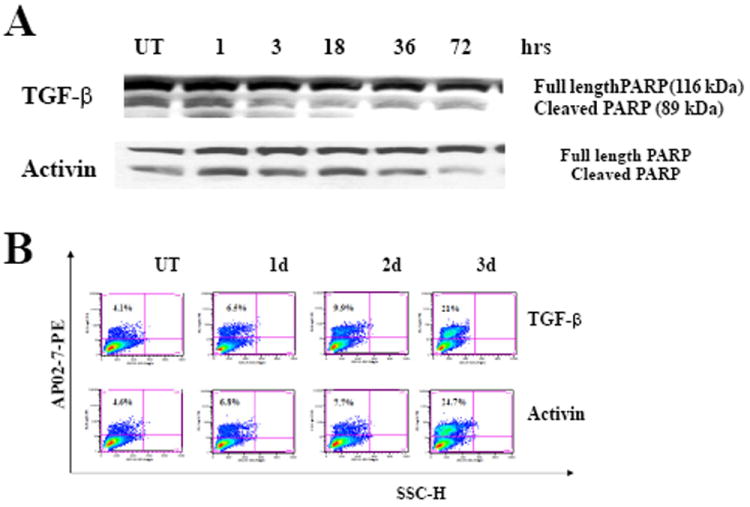
Apoptotic response of K562 cells to TGF-β1 and activin A treatment does not precede differentiation **(A)** K562 cells were treated with 5 ng/ml of TGF-β1 (top panel) and activin A (bottom panel) for 3 days. Time course analysis (as indicated) of PARP expression was performed using western blotting with an anti-PARP antibody that reacts with full length and cleaved PARP segments. **B**. Cells from the same cultures were evaluated for the expression of APO2.7 by flowcytometry at days 1, 2 and 3 using a PE-conjugated antibody against APO2.7. Similar results obtained in 3 independent experiments. Similar results were found using anti-phospho-Smad3. * *P*<0.05 and ** *P*<0.01.

Since the advent of treatment for acute promyelocytic leukemia (APL) [43] with all trans retinoic acid, differentiation therapy for treatment of leukemia has been a research focus. As TGF- and activin are multifunctional proteins that regulate growth, differentiation and apoptosis in various cell types [19,26,40], a relationship between activin/ TGF-β- induced erythroid differentiation/growth inhibition and the induction of apoptosis was examined. Lysates from cells treated with cytokines for different times until 72 hours, when 30-35% of TGF-β1 treated K562 cells were hemoglobinized by day 2, did not exhibit significant increases in the cleavage of PARP protein (Figure 4A), a specific marker of apoptosis in these cells [44]. The reversibility of this growth arrest mediated by activin/ TGF-β was examined. Cells were treated with 1-5 ng/ml of either agent for different periods of time, washed, recultured and their growth was compared to untreated control cells for up to 3 days. Exposure of cells to activin/ TGF-β for 2 days resulted in a reversible growth arrest (data not shown) and despite the growth inhibition of cytokine-treated cells for 2 days; cells remained largely viable and still began to accumulate hemoglobin. Moreover, FACS analysis of APO2.7, an early mitochondrial marker of apoptosis [39], at various times (1-3 days) after treatment with activin/ TGF-β revealed that a small fraction (<10%) of cells underwent apoptosis at day 2 (Figure 4B). At this time (day 2), we have noticed that the percent of Hb^+^ cells was 75% of the maximal percentage observed at the end of the culture (day 5) [45], indicating that cell apoptosis is not a feature of early differentiation. Therefore, activin/ TGF-β- mediated differentiation/growth inhibition is not preceded by the onset of apoptosis and that any delayed apoptotic response in cytokine treated cells (>20% by day3) (Figure 4B) may be the ultimate result of p38MAPK activation by activin/ TGF-β which is known to activate caspases during-terminal erythroid differentiation [36,37].

### Hydroxyurea and sodium butyrate induce Smad protein phosphorylation through a TGF-β type I receptor dependent mechanism during erythroid differentiation

**Figure 5:**
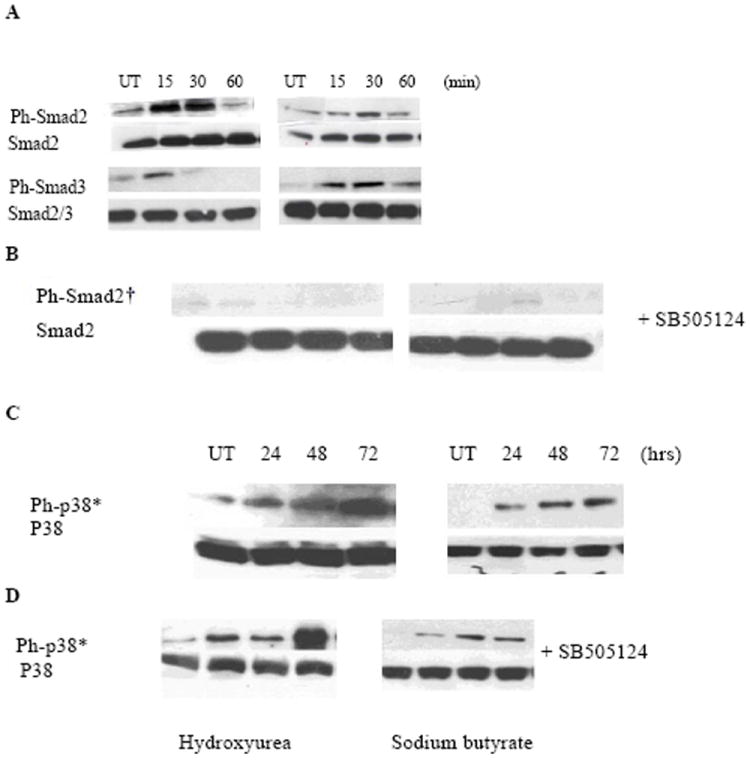
HU and butyrate stimulate phosphorylation of Smad2/3 and p38 MAPK. K562 cells were treated for indicated times with 400 M of HU (left) and 0.6 mM of sodium butyrate (right) without (A & C) and with (B & D) prior addition of SB505124 (20 μM). Membranes were immunoblotted with antibodies against phosphorylated Smad2, Smad3 and p38 MAPK proteins as shown in (A-D). The membranes were stripped and reprobed antibodies against total Smad2, Smad2/3, and p38 MAPK proteins (A-D). The data shown are representative of 3 independent experiments with similar results. Similar results were obtained using phospho-smad3 antibody.

**Figure 6:**
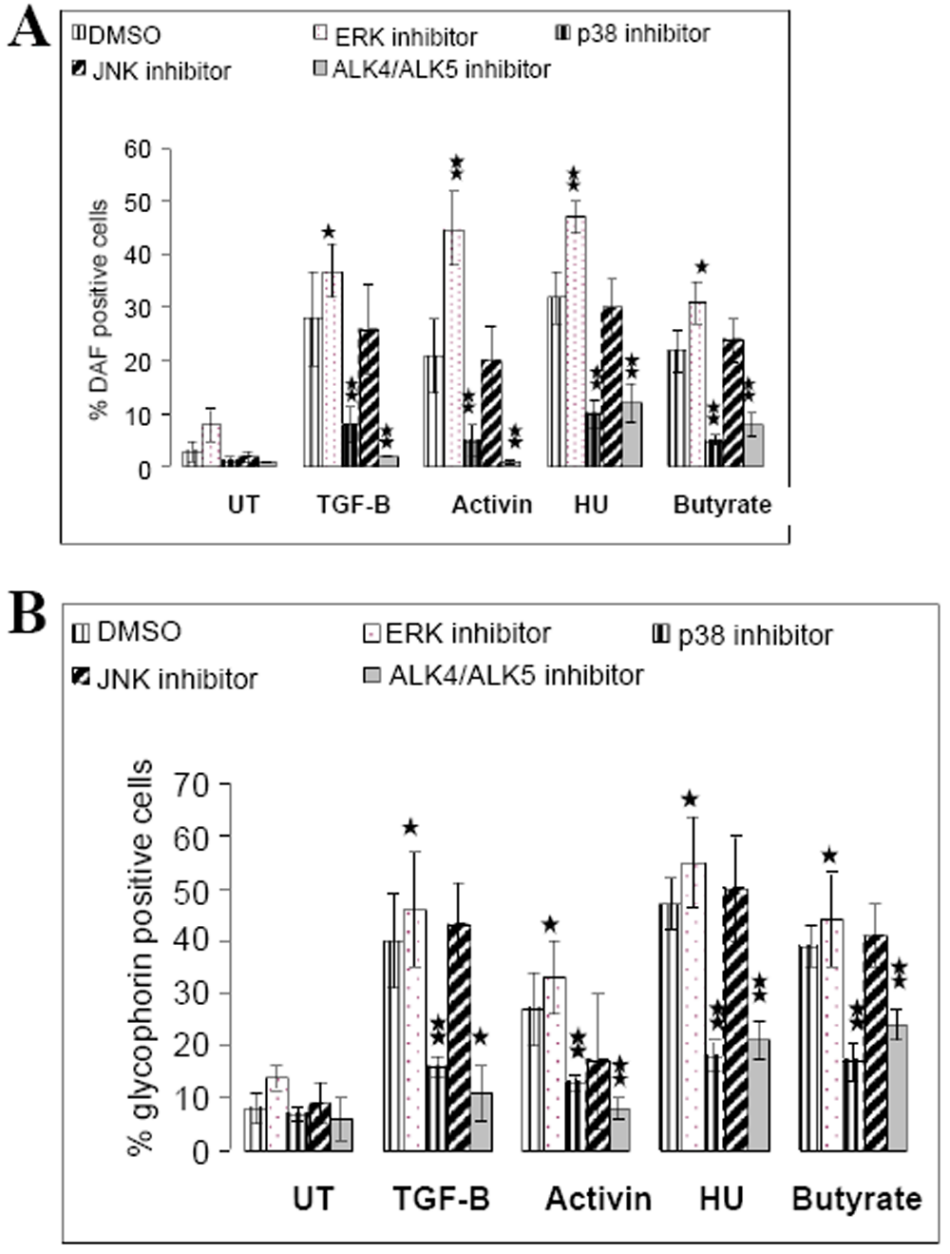
Inhibitors of MAPK or Smad signaling pathways block erythroid differentiation. **A**. K562 cells were pretreated for 1hr with and without the indicated inhibitors. Each culture was split into 5 groups. Groups were grown for 5 days in the absence and the presence of TGF-β1 (5 ng/ml), activin A (5 ng/ml), HU (150 M) and sodium butyrate (0.6 mM). Cell harvests were evaluated for the presence of Hb^+^ cells using DAF. **B**. K562 cells were pretreated for 1hr with and without the indicated inhibitors. Each culture was split into 5 groups. Groups were grown for 5 days in the absence and the presence of TGF-β1 (5 ng/ml), activin A (5 ng/ml), HU (150 M) and sodium butyrate (0.6 mM). Cell harvests were evaluated for the presence of Hb^+^ cells using DAF and for surface expression of GPA by flow cytometry. **B**. K562 cells were pretreated for 1hr with and without the indicated inhibitors. Each culture was split into 5 groups. Groups were grown for 5 days in the absence and the presence of TGF-β1 (5 ng/ml), activin A (5 ng/ml), HU (150 M) and sodium butyrate (0.6 mM). Cell harvests were evaluated for the presence of Hb^+^ cells using DAF and for surface expression of GPA by flow cytometry. *No increase in phospho-p38 was seen earlier than 24 hrs.

Since treatment with chemical inducers such as HU and sodium butyrate also stimulate Epo-independent hemoglobin synthesis in erythroleukemia cells similar to activin and TGF-β [30,34-35], the role of Smad signaling in mediating the effects of HU and butyrate was examined. Following the addition of HU (400M) and butyrate (0.6 mM) to serum-free cultures of K562 cells, both chemicals were able to induce a rapid, transient increase in phosphorylation of Smad2/3 at 15-30 min (Figure 5A), returning to base line by 60 min (by 30 min in hydroxyurea activation of Smad 3). These cellular treatments did not alter the amount of total Smad 2/3 proteins expressed in the cells, (Figure 5A). Moreover, similar regulation of phospho-Smad2/3 by HU and butyrate was found in other leukemic cell lines like TF-1 and HL60 (data not shown). Prior addition of the TβR1 signaling inhibitor, SB505124, to K562 cells treated with HU and butyrate abolished Smad2/3 phosphorylation (Figure 5B), and markedly reduced the development of erythroid (DAF^+^ and GPA^+^) cells (Figures 6A and 6B), compared with control cultures treated with DMSO (0.2%). These results indicate that HU and butyrate require TβR1-dependent signaling through Smads for erythroid differentiation.

### JNK, p38 and ERK MAPK proteins are transiently activated during activin/ TGF-β- induced erythroid differentiation

**Figure 7:**
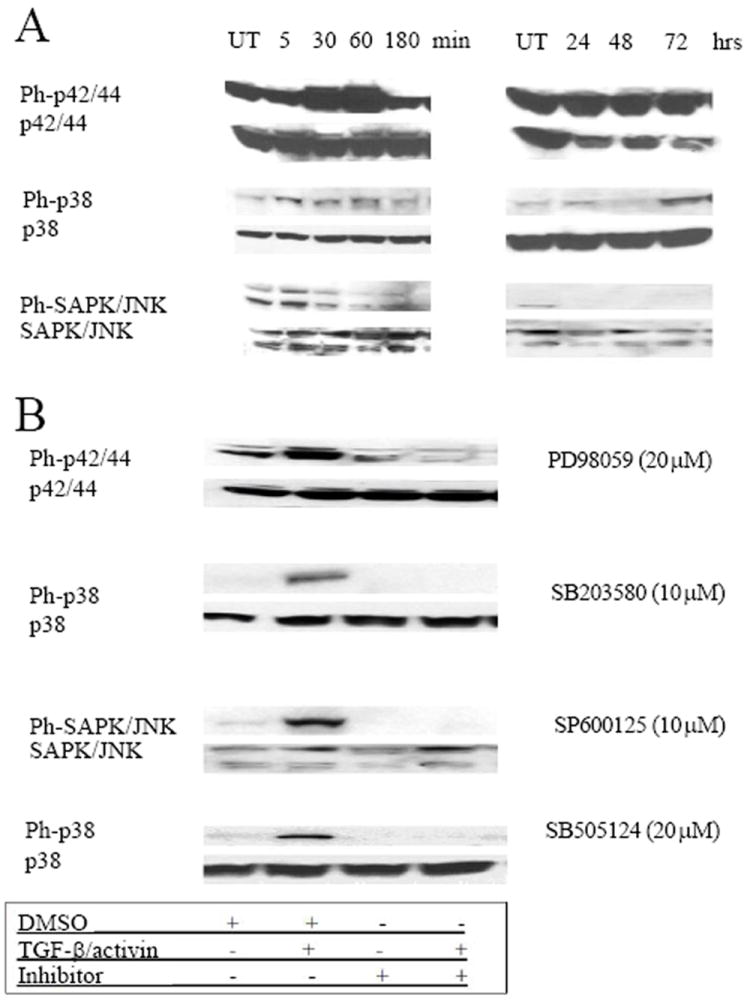
Phosphorylation pattern of the MAPK proteins induced by TGF-β and activin and inhibition of phosphorylation using specific inhibitors. **A**. K562 cells were treated with 5 ng/ml of TGF-β1, harvested at various time points (left: 5, 30, 60, and 180 minutes; right: 24, 48 and 72 hrs), lysed, and analyzed by immunoblotting. Membranes were probed with specific antibodies against the phosphorylated forms of the MAPK proteins (ERK1/2, p38 and SAPK/JNK), stripped and hybridized with antibodies against ERK1/2, p38 and SAPK/JNK. **B**. K562 cells were starved overnight and TGF-β or activin was added for 15-30 min with and without prior treatment with the signal transduction inhibitor. Inhibitors in were added as shown on the right of the panels. Phosphorylation of p42/44, p38 and SAPK/JNK was evaluated by western blotting as above. Results are representative of 3 independent experiments.

Similar to earlier reports, HU- and butyrate-induced differentiation of K562 cells was associated with activation of p38 (Figure 5C) and inactivation of ERK MAPK (data not 8 shown) [33-37]. Since TGF-β signaling can occur through the MAPK pathways [12-19], we investigated MAPK signaling in response to TGF-β/activin during erythroid differentiation. Unstimulated leukemic cells had basal levels of constitutively phosphorylated ERK1/2, p38 and JNK1/2 MAPK proteins despite being serum deprived overnight (Figure 7A) and stimulation by both cytokines revealed similar, rapid, transient changes in phosphorylation of all MAPK proteins studied (Figure 7A). Specific inhibitors of the MAPK proteins were effective in blocking TGF-β mediated MAPK signaling (Figure 7B) and inhibition of one MAPK pathway did not alter the phosphorylation of other tested MAPKs (data not shown), thus MAPK pathways including ERK, p38 and JNK send independent signals in K562 in response to TGF-β/activin stimulation. Prior treatment of TGF-β/activin-stimulated cells with SB505124 prevented the induction of phosphorylation of p38 and JNK MAPKs (Figure 7B), suggesting that these effects on MAPK kinases induced by TGF-β/activin are mediated by TβR1 signaling. Prior treatment of the TβR1 signaling inhibitor on HU/butyrate-induced MAPK activation indicated that SB505124 reduced but neither abolished p38 MAPK activation (Figures 5C and 5D) nor reversed cell growth inhibition (data not shown), suggesting that regulation of p38 MAPK by HU and butyrate is partially independent of TβR1 receptor signaling.

### Crosstalk between the ERK MAPK and Smad pathways regulate erythroid differentiation

**Figure 8:**
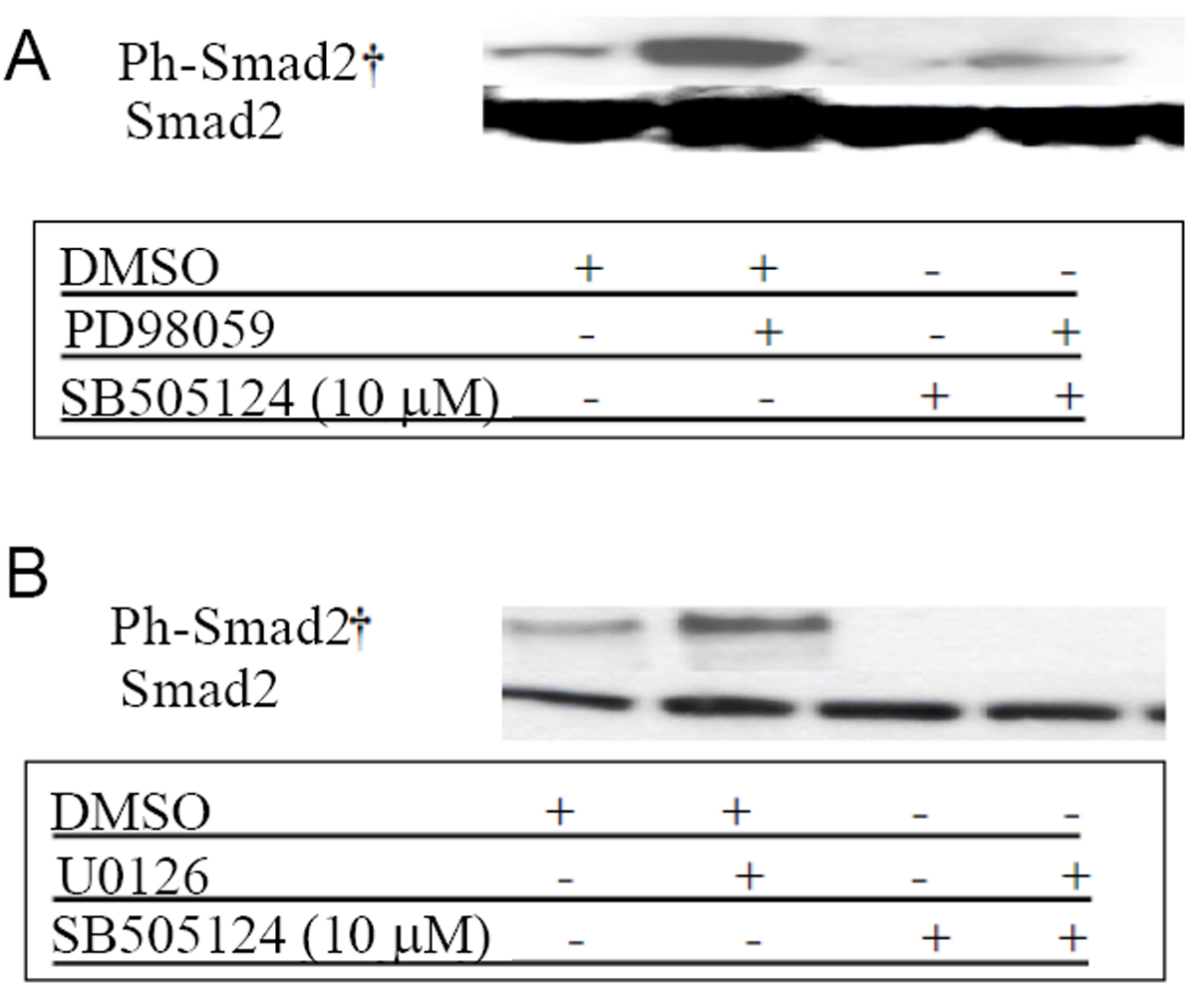
ERK1/2 MAPK inhibitors increase phosphorylation of Smad2/3 in presence of TGF-β receptor signaling. **A**. K562 cells were treated for 1 hr with PD98059 (20 μM) or U1026 (10 μM). **B**. In the presence and absence of the inhibitor of TGF-β type I receptor, SB505124 (20 μM), as indicated. Phosphorylation of Smad2/3 and expression of endogenous Smad2/3 were evaluated by western blotting. Results are representative of 3 independent experiments. Similar results were obtained using antibodies against phophorylated ERK1/2 and SAPK/JNK proteins.

**Figure 9:**
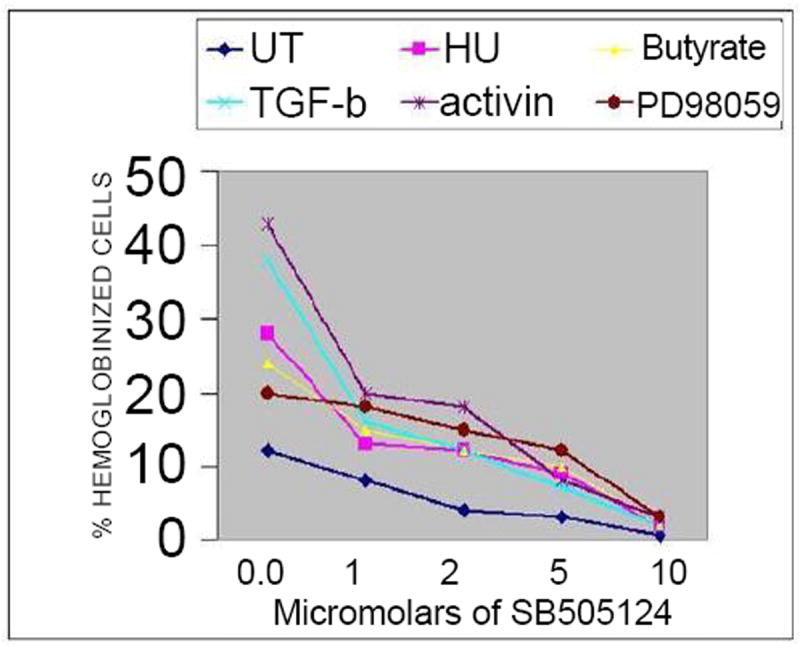
Dose response of TGF-β receptor signaling inhibitor on induction of erythroid differentiation. K562 cells were pretreated for 1hr with and without the indicated concentrations of SB505124 inhibitor. Each culture was split into 6 groups. Groups were grown for 5 days in the absence and the presence of TGF-β1 (5 ng/ml), activin A (5 ng/ml), HU (150 M), sodium butyrate (0.6 mM) and PD980059 (10 uM). Cell harvests were evaluated for the presence of Hb^+^ cells using DAF. Results are representative of 3 experiments. Same results were obtained using anti-phospho-Smad3 and Smad2/3 antibody.

Sustained activation of ERK MAPK is required for megakaryocytic differentiation of erythroleukemia cells [45,46] and inhibition of ERKMAPK leads to erythroid differentiation [33-37]. Since ERK MAPK activation can down regulate Smad phosphorylation and Smad 7 can block erythroid differentiation and stimulate megakaryocytic differentiation in K562 cells [47,41]. ERK could mediate these effects through Smad signaling. The ERK1/2 MAPK pathway was transiently activated, after 5 to 60 minutes of cytokine treatment, returned to basal level by 3 hrs, and remained at basal level erythroid differentiation (24-72 hrs), (Figure 7A). A 1 hr pretreatment with an ERK inhibitor (PD98059), prior to cytokine stimulation deregulates ERK activation (Figure 7B) and significantly (P<0.05) synergizes with TGF-β/activin in erythroid differentiation (Figure 6). Prior addition of PD98059 elicited a similar effect on the differentiation of K562 cells treated with HU and butyrate, suggesting that ERK MAPK negatively regulates erythroid differentiation induced by all tested agents. Treatment of cells with the inhibitors of ERK1 (PD98059 10 μM) and ERK1/2 (U1026, 10 μM) alone resulted in a robust phosphorylation of Smad2/3 (Figure 8) and induced a slight increase in the proportion of erythroid (DAF^+^ and GPA^+^) cells (Figure 6). Prior addition of SB505124 prevented ERK inhibitor-induced Smad2/3 phosphorylation (Figure 8) and erythroid differentiation in a dose dependent manner (Figure 9). Hence, ERK MAPK negatively regulates Smad-mediated erythroid differentiation through TRI-dependent signaling. The ability of an inhibitor of TβR1 receptor-mediated signaling to block the erythroid differentiation of these differentiation inducers (Figure 9) indicates that TGF-β signaling is critical element needed for this differentiation.

### Activation of JNK MAPK does not modulate TGF-β-, activin-, HU- and butyrate- induced erythroid differentiation

We observed here that treatment with TGF-β and activin activates JNK1/2 MAPK (Figure 7A). Using a specific inhibitor for JNK1/2/3 (SP600125, 10 μM) [48], we were able to block JNK1/2 activation induced by TGF-β/activin (Figure 7B). Inhibition of JNK1/2 MAPK activation had no significant effect (P>0.05) in the formation of DAF^+^ and GPA^+^ cells by TGF-β, activin, HU or butyrate (Figure 6). Thus, it appears that activation of JNK/1/2 is not involved in the regulation of terminal erythroid differentiation of erythroleukemia cells.

### Erythroid differentiation of erythroleukemia cells by activin, TGF-β, HU and butyrate involves activation of p38 MAPK

Activation of the p38 MAPK pathway was found to be critical for Epo-, butyrate-, and HU- induced hemoglobin synthesis [33-37]. Our data confirmed this relationship between the p38 MAPK pathway and TGF-β/activin in differentiation of K562 cells. Cytokine treatment caused transient activation of p38 MAPK (Figure 7A), and specific inhibition of p38 MAPK by SB203580 (10 M) prevented p38 phosphorylation (Figure 7B), and abrogated the appearance of DAF^+^ and GPA^+^ cells (Figure 6). Moreover, prior treatment of TGF-β/activin-stimulated cells with SB505124, which interferes with ligand/receptor signaling, prevented the activation of p38 MAPK and erythroid differentiation (Figure 9). Thus, p38 MAPK activation is involved in TGF-β/activin-induced erythroid differentiation.

We have observed high levels of p38 MAPK phosphorylation after 72 hrs of cytokine stimulation (Figure 7A). Similar levels of phospho-p38 MAPK were found in cells treated for more than 24-48 hrs with HU and butyrate (Figure 5C). Addition of p38 MAPK inhibitor up to 36 hrs after cell treatment with HU, butyrate, activin or TGF-β caused an inhibition of erythroid differentiation the same as when added prior to cell treatment (data not shown), which raises the probability that p38 MAPK activation is a late but essential event in erythroid differentiation. The activation of capases by p38 MAPK suggests that it is involved the apoptosis seen in late stage differentiation [35-37].

### Activation of Smad2/3 and p38 MAPK by the protein phosphatases inhibitor okadaic acid alone induces erythroid differentiation

**Figure 10:**
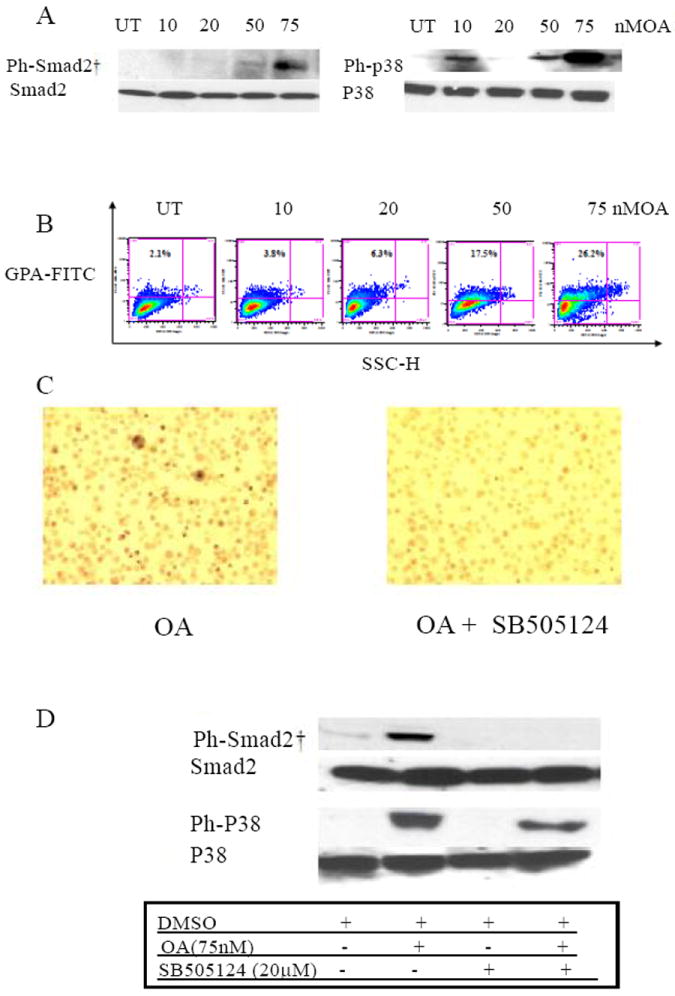
Induction of activation of Smad2/3 and p38 MAPK, and erythroid differentiation in K562 cells treated with Okadaic acid. Cells were starved, treated with various concentrations (10-75 nM) of OA for 3 hrs then incubated with 2.5 nM OA. Similar results were found using anti-phospho-smad3.

We have previously shown that okadaic acid (OA) treatment results in phosphorylation of Smad2 in HL60 cells and promotes monocytic differentiation [40]. OA is an inhibitor of the serine/threonine protein phosphatases, which can upregulate the phosphorylation of signaling intermediates and alter cell fate in the absence of added differentiation agents. Starved K562 cells were treated with various amounts of OA for 3 hrs, washed 3 times and cultured in the presence of 5% FBS and 2.5 nM of OA. Treatment of cells with 75 nM OA alone resulted in a marked increase in phospho-Smad2/3 and -p38 MAPK (Figure 10A). This change in the signaling balance by OA alone was enough to increase the proportion of cells expressing GPA (Figure 10B) and accumulating intracellular Hb (Figure 10C) in a dose dependent fashion. Consistent with this, OA enhanced erythroid differentiation induced by activin, TGF-β, HU and butyrate (data not shown). Pre-treatment of the cells with the TR I receptor signaling inhibitor, SB505124, selectively prevented OA-induced phosphorylation of Smad2/3 but only had a marginal effect on p38 MAPK activation (Figure 10D) and resulted in subsequent inhibition of formation of Hb^+^ cells (Figure 10C) and GPA^+^ cells (data not shown). These findings emphasize that activation of p38MAPK alone is not enough to promote erythroid differentiation of K562 cells, which requires activation of Smad2/3 and p38MAPK pathways.

## Discussion

A distinct role for TGF-β and activin in erythropoiesis has been reported in studies of primary and transformed cells [16-28,30-32,36,49,50]. However, given the many stages of erythropoiesis from the stem cell quiescence to red cell enucleation and the many cytokines involved, these studies cannot be fully informative about the context in which signaling networks co-mediate TGF-β/activin effects [8,9]. In terms of leukemia, since the success of treating APL with retinoic acid [43], there has been interest in developing differentiation therapies. To study these two problems, erythroleukemia cell lines, which have the potential to give rise to erythroid and other mature hematopoietic lineages were used as a model to study the changes necessary for erythroid differentiation. Three classes of inducers were used: 1) cytokines, TGF-β and activin, 2) chemicals, HU and butyrate, and 3) a phosphatase inhibitor, OA. We have delineated the importance of cross-talk between receptor-activated Smad signaling and the MAPK pathways in the regulation of erythroid differentiation and growth inhibition seen with all of these inducers. Treatment of cells with TGF-β/activin resulted in inhibition of cell growth and led to erythroid differentiation as evidenced by a significantly increased proportion of Hb-containing cells. Changes in cell fate were preceded by cytokine/ receptor-mediated intracellular signaling, which involved activation of the receptor-activated Smads; Smad2 and Smad3, and various cascades of MAPK; ERK, p38 and JNK. Direct activation of Smad2/3 by TGF-β type I receptors is well established [1-4] as well as studies describing links between MKK4/JNK and MKK3/p38 activation and TβR1 signaling involving XIAP, HPK1 and TAK1 [1-4,12-19]. Active ERK MAPK may negatively or positively modulate receptor mediated Smad activation and nuclear translocation [18,20-22]. Inhibition of TβR1 signaling abrogated TGF-β/activin-induced activation of ERK, p38 and JNK MAPKs in K562 cells. This suggests that all these MAPKs may actually be linked to TβR1-mediated Smad signaling during erythroid differentiation. Our results are in accordance with the results, showing that SB505124 interferes with TGF-β-induced activation of MAPKs [42].

There is a strong correlation between leukemic transformation and loss of sensitivity to the antiproliferative effects of TGF-β, which is frequently associated with defective Smad signaling [51,52]. The effects of TGF-β in various erythroleukemia cells showing association between Smad signaling, and suppression of cell growth and erythroid differentiation suggests that the Smad pathway is intact in these transformed cells. In K562 cells that represent leukemic human hematopoietic progenitor cells (HPC), we have explored the role of the TGF-β/activin-induced signaling in mediating cell growth arrest. TGF-β/activin-induced growth arrest was reversed in cells pretreated with the inhibitor of TGF-β/activin type I receptors and to a lesser extent with a p38 MAPK inhibitor but not with ERK inhibitors. Also, activation of p38 MAPK mediates growth inhibition of normal HPC by TGF-β [53]; thus the suppressive effect of TGF-β in normal and leukemic human HPC involves activation of both Smad2/3 and p38MAPK.

Further evidence of cooperation between these two pathways was seen in the Epo-independent erythroid differentiation by chemicals. Cytokine-induced erythroid differentiation was dependent on the activation of p38 MAPK. Either selective inhibition of p38 by SB203580 or co-inhibition of phosphorylation of Smad2/3 and p38 MAPK by SB505124 was sufficient to prevent the formation of GPA^+^ Hb^+^ cells. Because it was not possible to achieve selective inhibition of phosphorylation of Smad2/3 without inhibition of p38 MAPK by SB505124 in cells treated with TGF-β/activin, it remains uncertain whether activation of Smad2/3 is a prerequisite for TGF-β/activin-induced erythro-differentiation. We and others have reported that overexpression of Smad7, which interferes with TRI receptor activation of Smad2/3 phosphorylation and inhibited erythroid differentiation induced by TGF-β or activin [41]. We found that ectopic stable expression of Smad7 also prevented phosphorylation of p38 MAPK in response to TGF-β/activin treatment [45], clouding the requirement of phospho-Smad2/3 in TGF-β/activin-induced erythroid differentiation.

We have previously shown that okadaic acid stimulates phosphorylation of Smad2 in HL60 cells and promotes monocytic differentiation [40]. Okadaic acid-induced Smad2/3 and p38 MAPK activation in K562 cells resulting in the induction of erythroid differentiation and promotion of differentiation induced by various other agents. The selective inhibition by SB505124 of phosphorylation of Smad2/3 but not p38 MAPK in OA treated cells was sufficient to prevent OA-induced differentiation. Thus, activation of p38MAPK alone is insufficient for erythroid differentiation and activation of Smad2/3 is also required. This ability of TGF-β signaling through the TβR1 receptor to activate both Smad and MAPK pathways ensure their cooperation in differentiation pathways [54,55]. HU and butyrate induce cytodifferentiation and growth inhibition of a variety of tumor cells, and have been used in cytoreductive and differentiation therapy of malignant disease [56-58]. The ability of these chemicals to alter gene expression is mediated in part by changes in signal transduction pathways. Butyrate caused sustained activation of JAK/ STAT signaling in murine erythroleukemia cells [59]; erythro-differentiation of K562 by HU and butyrate involved the phosphorylation of p38 and dephosphorylation of ERK MAPKs [33-37]. In this report, we have shown that the canonical signaling pathway (Smad activation) for the TGF-β family is also involved in the regulation of HU- and butyrate-induced erythroid differentiation of K562 cells. Although, no direct binding has been described between HU or butyrate and TGF-β receptors, results of SB505124 indicate that both chemicals activate the TGF-β type I receptor kinase upstream of Smad2/3. It remains unclear whether these chemicals act directly or indirectly (through autocrine TGF-β) to regulate receptor mediated Smad signaling. Attempts to block ligand induction of differentiation by anti- TGF-β antibody did not prevent HU/butyrate-induced erythroid differentiation (data not shown). Given that these agents induce phosphorylation of Smads so quickly, it is possible that they, like OA, block phosphatase activity which increases the strength of basal endogenous signaling by autocrine TGF-β/activin in K562 cells leading to erythroid differentiation. The biological effects of HU and butyrate on erythro-differentiation, growth inhibition, cell cycle arrest at G1 phase, and stimulation of fetal hemoglobin synthesis share similarity with those of TGF-β [60,61], supporting the idea that these agents may mediate their effects through common signal transduction pathways. Collectively our data suggest that activation of both Smad2/3 and p38 MAPK is required during erythroid differentiation.

For erythro-megakaryocytic differentiation several reports demonstrate that ERK MAPK negatively regulates erythroid differentiation [33-37] and that sustained activation of ERK MAPK is sufficient to induce a differentiation program along the megakaryocytic lineage [41,45-47]. Consistent with these reports, we have shown that ERK1/2 is transiently activated by TGF-β/activin but reduced below basal level during erythroid differentiation. Moreover, inhibitors of ERK1/2 enhanced erythroid differentiation in the absence and the presence of TGF-β/activin stimulation. Similar synergism was reported between ERK inhibitors and HU and butyrate, showing that ERK MAPK negatively regulate this Epo-independent hemoglobin synthesis [34,35]. We found that ERK inhibitors induced Smad2/3 phosphorylation, which was dependent on TRβ1 signaling. Similar results were seen in HL60 and TF-1 cells (data not shown), indicating that crosstalk between the Smad and ERK MAPK pathway is a modulator of cell differentiation. Inhibition of ERK MAPK in mouse embryo fibroblasts potentiates Smad signaling and activation of Smad-dependent gene targets [62]. Also, ERK inhibition increased both the basal and TGF-β-induced Smad7 promoter activity in rat fibroblasts [63]. Based on these findings, inhibition of ERK MAPK augments Smad2/3 signaling, which by itself leads to increase Smad7 transcripts as part of the negative feedback loop of TGF-β/Smad signaling [1-7,41].

Antisense oligonucleotides of JNK1 and JNK2 suppressed Epo induced hemoglobinization in SKT6 cells [33]. We showed an activation of JNK1/2 in K562 cells treated with TGF-β, but use of a specific JNK inhibitor, SP600125, did not influence the formation of Hb^+^ cells. Similarly, the JNK1/2/3 inhibitor had no effect on hemoglobinization induced by HU and butyrate. Thus it seems that activation of JNK is dispensable during the development of erythroid cells by HU, butyrate and TGF-β/activin.

In summary, our data provide evidence that both TGF-β Smad and p38 MAPK signaling networks are needed during the Epo-independent erythrodifferentiation of erythroid leukemia cells stimulated with TGF-β, activin, HU, OA and butyrate. We showed that signals through the Smad and the p38 MAPK pathways are independent but integrate in erythroid differentiation while the balance between the interrelated Smad and ERK MAPK signals appear to also play a role in the ultimate differentiation outcome.
